# The Role of Actin Dynamics and Actin-Binding Proteins Expression in Epithelial-to-Mesenchymal Transition and Its Association with Cancer Progression and Evaluation of Possible Therapeutic Targets

**DOI:** 10.1155/2018/4578373

**Published:** 2018-01-16

**Authors:** Magdalena Izdebska, Wioletta Zielińska, Dariusz Grzanka, Maciej Gagat

**Affiliations:** ^1^Department of Histology and Embryology, Faculty of Medicine, Nicolaus Copernicus University in Toruń, Collegium Medicum in Bydgoszcz, Bydgoszcz, Poland; ^2^Department of Clinical Pathomorphology, Faculty of Medicine, Nicolaus Copernicus University in Toruń, Collegium Medicum in Bydgoszcz, Bydgoszcz, Poland

## Abstract

Metastasis causes death of 90% of cancer patients, so it is the most significant issue associated with cancer disease. Thus, it is no surprise that many researchers are trying to develop drugs targeting or preventing them. The secondary tumour site formation is closely related to phenomena like epithelial-to-mesenchymal and its reverse, mesenchymal-to-epithelial transition. The change of the cells' phenotype to mesenchymal involves the acquisition of migratory potential. Cancer cells movement is possible due to the development of invasive structures like invadopodia, lamellipodia, and filopodia. These changes are dependent on the reorganization of the actin cytoskeleton. In turn, the polymerization and depolymerization of actin are controlled by actin-binding proteins. In many tumour cells, the actin and actin-associated proteins are accumulated in the cell nucleus, suggesting that it may also affect the progression of cancer by regulating gene expression. Once the cancer cell reaches a new habitat it again acquires epithelial features and thus proliferative activity. Targeting of epithelial-to-mesenchymal or/and mesenchymal-to-epithelial transitions through regulation of their main components expression may be a potential solution to the problem of metastasis. This work focuses on the role of these processes in tumour progression and the assessment of therapeutic potential of agents targeting them.

## 1. Introduction

Actin is a highly conservative protein commonly occurring in eukaryotic cells. In nonmuscle cells, actin represents 1–5% of all cell proteins, whereas for muscle cells it can be even up to 10%. In eukaryotic cells, we may distinguish two main actin forms: globular G-actin and fibrillar F-actin. The globular form is a 43 kDa monomer, while the fibrillar is a long-chained polar polymer resulting from the G-actin polymerization [1]. The globular form is spread evenly between cytoplasm and nucleoplasm; the distribution of F-actin filaments depends on the cell type, its role, and the cell cycle phase in which it is located [2–6]. Reorganization of the actin structure and the transition between its forms play a significant role in many of the most important cellular processes like cytokinesis, cell differentiation, and death [6–8]. The presence of fibrillar actin has been observed in both suspension and adherent cell lines. It was proved that F-actin occurs in form of short fibres in the leukemia cells cytoplasm [9–11] while in the case of adherent cells it was observed for example as stress fibers [12]. Both actin forms also play a role in pathological events like cancer transformation [13] or cells' response to the negative effects of external factors like mechanical stress [14] or chemicals [12]. Undeniable evidence that fibrillar form of actin can be visualized using phalloidin in stress conditions such as cytostatic drugs induction or heat shock was presented by Grzanka et al. [15, 16]. As a result of cell death induction round structures of F-actin were observed inside the cell nucleus. This effect occurred in the sequences poorly stained with DAPI and therefore probably in the DNA regions rich in G-C pairs with high transcriptional activity [17].

For many years scientists questioned the presence of F-actin in the cell nucleus, however as it was proven by McDonald et al. polymeric and monomeric actin forms can be observed in the nucleus by photobleaching [18]. Additionally, Izdebska et al. presented a simple, phalloidin-based method of F-actin detection at the TEM (transmission electron microscope) level using streptavidin-conjugated CdSe/ZnS semiconductor quantum dots and the combination of pre- and postembedding techniques [19], although it is still unclear whether its presence is continuous or only periodical. Nevertheless, many studies indicate the important role of actin in processes occurring in the cell nucleus [20–22]. Nuclear actin is proved to be a part of chromatin remodelling complexes and transcription machinery; it is also included into newly synthesized ribonucleoproteins [23]. In addition, it has an impact on long-range chromatin organization, nucleocytoplasmic transport, and nucleus structure [21, 22, 24]. The contribution of nuclear actin in chromatin remodelling during active cell death has been confirmed by the SATB1 (special AT-rich sequence-binding protein-1) and F-actin colocalization in transcriptionally active regions of the nucleus. It has also been shown that interactions between the SATB1 and the densely packed F-actin are present at the boundary between condensed and noncondensed chromatin regions [6, 25].

## 2. Transport of Actin in and out of a Nucleus

**Figure 1:**
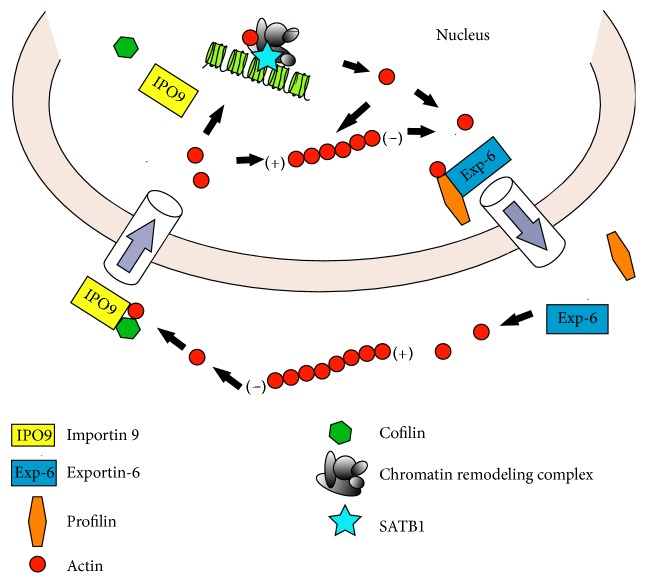
*Transport of actin in and out of the cell nucleus*. Actin is imported into the nucleus in complex with cofilin by nuclear import factor, importin 9. Actin is exported outside the nucleus in complex with profilin by nuclear export factor, exportin-6.

Although actin does not contain a classical NLS (nuclear localization sequence), it is still likely that it penetrates from the cytoplasm to the nucleus due to translocation, as many ABPs (actin-binding proteins) comprise NLS [26]. One of these proteins is CFL-1 (cofilin-1), which was proved to be responsible for the accumulation of actin in the cell nucleus in a response to stress conditions [27]. It has been shown that, due to doxorubicin-induced cell death in the case of the CHO AA8 cell line, change in the expression of nuclear cofilin occurs. This phenomenon was associated with the increased value of actin translocation from the cytoplasm to the nucleus [28]. However, it has not been clarified yet whether this process takes place under physiological conditions [27]. Grzanka et al. also demonstrated that the downregulation of cofilin-1 during doxorubicin treatment results in suppression of apoptosis and induction of cell death primarily through mitotic catastrophe. They concluded that the mitotic catastrophe as a cell death mechanism is independent of F-actin while cofilin is a potential apoptosis inducer [12]. In 2012 Dopie et al. presented another factor involved in actin translocation to the nucleus, importin 9 (IPO9) [29]. Afterwards, Izdebska et al. described the correlation between downregulation of IPO9 and the statistically significant decrease in the percentage of apoptotic cells as well as the increase in actin content in both cytoplasm and cell nucleus of MCF-7 cells. It was also associated with the increase in CFL1 expression. The obtained results suggest that CFL1 is not the only essential factor for effective actin translocation to the nucleus as this process also requires the presence of IPO9 [30]. On the other hand, the export of actin from a nucleus to cytoplasm usually requires nuclear transport receptors from *β*-importin superfamily. One of the representatives is Exp-6 (exportin-6), which in the complex with profilin participates in the transition of actin from the nucleus [31]. It has also been proved that the downregulation of Exp-6 expression in H1299 cells results in cell adhesion abnormalities, indicating the important role of accurate actin distribution between cytoplasm and nucleus in the preservation of proper cell adhesion [32] (Figure 1).

## 3. Epithelial-Mesenchymal Transition and Its Connection with Intracellular Actin Organization

The studies presented above point to the important role of actin and its structural changes in both cell survival and active death. However, the connection between this protein and the occurrence of EMT (epithelial-mesenchymal transition) has also been confirmed. EMT was first described in early 80s by Greenburg and Hay as a process in which epithelial cells acquire characteristics of mesenchymal phenotype and lose their connections to neighbouring cells [33]. This phenomenon plays an important role in the embryo implantation and initiation of the placenta formation [34]. In a response to growth factors such as TGF-*β* (transforming growth factor *β*) or EGF (epithelial growth factor) EMT is induced through activation of Wnt and Notch signalling pathways, which causes downstream activation of such transcription factors as Snail, ZEB, Smad, and Twist [13]. In the next stage E-cadherin (epithelial cadherin) is degraded, which results in plasma membrane disintegration and breakage of interaction with *β*-catenin; simultaneously the expression of the main EMT markers, N-cadherin (neural cadherin) and vimentin, rises [35]. As a result of EMT epithelial cells lose their polarity and junctions, while gaining migratory potential and invasiveness. Changes in the structure of the cytoskeleton and the expression of genes responsible for cell shape can also be observed [35].

One of the main features of epithelial cells is their integrity, which is maintained by the presence of several intercellular connections types like tight junctions, adherens junctions, and desmosomes [36]. The essential element in both stabilization and regulation of junctions functioning is interactions of adhesion proteins with actin [37]. The connection of adhesion proteins with cytoskeleton components and signalling molecules is possible due to the presence of cytoplasmic tails. Their bonding allows the intracellular transport of molecular signals [38–41]. Harris et al. also confirmed the relationship between actin filaments and cadherins and indicated their key role in AJs (adherens junctions) creation [42, 43]. It was also shown that, in early stages of the EMT, AJs are destroyed by the degradation or displacement of junction proteins. This is associated with decrease in claudin and occludin expression [36]. The entire process of transition from the epithelial-to-mesenchymal phenotype must be proceeded by strict control of cell motility programs by regulating gene expression, posttranslational modification of proteins, and reorganization of the cytoskeleton. Changes in the organization of cortical actin cytoskeleton occurring in the course of EMT result in gaining of fibroblastic morphology by cells, which in turn allows the polarized assembly of the cytoskeleton to form protrusive and invasive structures. That creates the possibility of cell elongation and changes directional motion dynamics [44].

**Figure 2:**
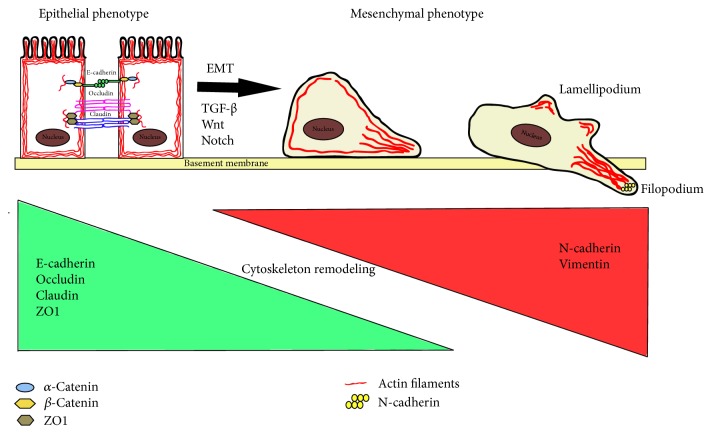
*The most important features of epithelial-to-mesenchymal transition (EMT)*. During EMT downregulation of E-cadherin, occluding, claudin, and ZO1 and upregulation of N-cadherin and vimentin occur.

Due to the varied effects of the EMT process, it was classified into three types. The first one is type-1 EMT, which occurs during embryogenesis, the second is type-2 EMT that can be observed in the course of wound healing, and the third is type-3 EMT, which contributes to tumour progression and metastasis [35] (Figure 2).

In terms of intracellular signalling type-1 EMT is inseparably connected with the Wnt pathway. Embryos with Wnt3 deficiency cannot undergo EMT connected with gastrulation [45], while Wnt8c ectopic expression results in multiple primitive streaks [46]. Moreover, deficiencies in some of Wnt pathway mediators like Nodal and Vg1 were also associated with lack of functional EMT [47, 48]. In turn, type-2 may be triggered by release of growth factors such as EGF, PDGF, FGF-2, or TGF-B near the injury site [49, 50]. Gradient of growth factors along with the chemoattractants produced by the immune system cells causes the transition epithelial cells migration to the wound in order to remove a damage. In the case of type-3 EMT, growth factors such as HGF, EGF, PDGF, and TGF-*β* are also involved. However, this process is more complicated and includes the involvement of transcription factors, Snail, Slug, and ZEB1 (zinc finger E-box binding homeobox 1), as well as other factors, *β*-catenin, actin, ABPs, or Ras proteins [35]. Some of them are described in more detail in the next section of the article.

## 4. Role of Actin Dynamic in EMT and Its Association with Metastasis and Cancer Progression

**Figure 3:**
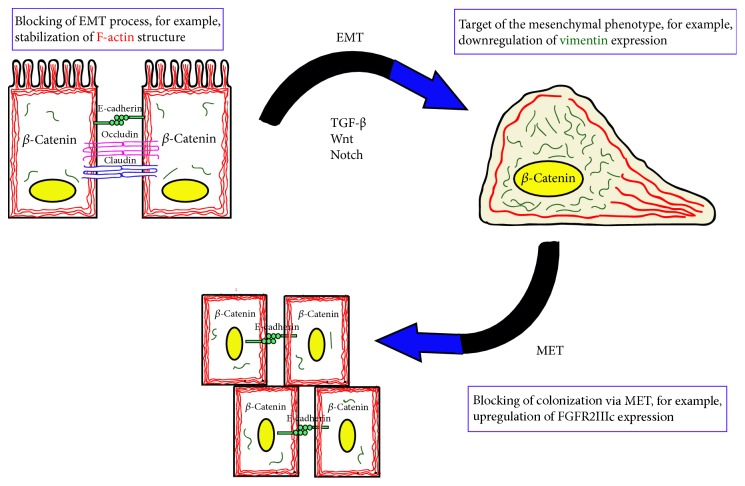
*Main changes in β-catenin and E-cadherin expression during EMT and MET with possible therapeutic targets*. During EMT process *β*-catenin expression pattern changes from cytoplasmatic to nuclear; simultaneously E-cadherin is downregulated and vimentin is upregulated. After MET process occurs expression pattern again turns to epithelial.

The epithelial-mesenchymal transition in the course of a cancer is generally associated with negative effects and worse prognosis. EMT increases the migratory potential and invasiveness of abnormal cells, which increases the risk of metastasis [51]. Perl et al. proved that in transgenic mouse model of pancreatic *β*-cell carcinogenesis the change of phenotype from a well-differentiated adenoma to invasive carcinoma is associated with the loss of E-cadherin expression [52]. Also in the case of breast cancer hypoxia-induced decrease in expression of E-cadherin correlated with an increase in the migratory potential of cells [53]. Another example may be the increased expression of the Notch-1 protein in bone metastases of prostate cancer in comparison to the primary tumours [54]. In addition, in colorectal cancer, the loss of membranous *β*-catenin led to tumour cell budding, a morphological marker of aggressiveness and invasiveness of this cancer cell type [55]. However, the fact that in metastases also mainly cells with epithelial phenotype were found remains problematic. The explanation may be the occurrence of reverse to EMT process, MET (mesenchymal-epithelial transition) at the site of secondary tumour formation [56]. MET is again associated with changes in E-cadherin expression and proliferative activity. Brabletz et al. have proved that well-differentiated central tumour cells of primary colon cancer exhibit only cytoplasmic *β*-catenin expression, which coincides with the pattern of expression in healthy colon epithelial cells. With the acquisition of tumour cell invasiveness, they begin to exhibit strong nuclear expression of *β*-catenin, which is not observed in the well-differentiated metastatic cells [51, 57]. These evidences suggest the occurrence of rechange in the phenotype of the cells in the metastasis site (Figure 3). This stage of cancer progression can also be the target of potential drugs [58]. Another issue connected with EMT is the presence of CSCs (cancer steam cells), which stands for the subpopulation of tumour cells capable of forming secondary tumours. It was suggested that transformation of non-CSC to CSC requires EMT [59].

Ability to metastases creation is one of the most important features acquired by the cancer cells contributing to tumor progression. This requires movement of cells from a primary tumour to a new habitat. This process is closely related to EMT and consists of several steps. The first one is the removal of the cell from a primary tumour, which is followed by intravasation of the blood vessels. The next stage is extravasation and implantation of the cell in another body compartment. The last step is cell proliferation leading to the formation of a secondary tumour [60]. Cell migration is facilitated by the polymerization and depolymerization of actin. The polymerization is connected with the formation of membrane projections such as sheet-like membrane protrusions, lamellipodia, or spike-like extensions at the edge of lamellipodia called filopodia [61]. This was confirmed by a study using growth factors to stimulate cell migration. As a result of EGF activity, lamellipodia was formed after just a few minutes [62]. Another study showed that in the lamellipodium structure amount of the polymerized actin is approximately 3.3 times higher than the monomeric form [63]. Abnormal expression of growth factors in case of cancer leads to excessive cell proliferation and migration [64, 65] and has been found in such cancers types as colorectal carcinoma [66] or glioma [67]. In addition, an increased level of growth factors in the serum has been associated with higher risk of lung [68] and prostate [69] cancer. Actin also contributes to the survival of tumour cells during transport in blood vessels, as it protects them from degradation and makes productive connections with blood cells such as erythrocytes or thrombocytes, which prevents cancer cells from the immune system action [61]. The next step, extravasation, also requires actin's presence. Escape from the vasculature involves attaching the cell to the endothelium, passing through the vascular wall, and ECM (extracellular matrix) to establish a new tumour site. ECM penetration is possible thanks to actin-rich dynamic protrusions invadopodia, which exert a proteolytic function in ECM degradation allowing cells to enter into new environments [70]. Finally, in case of EMT increased cell contractility and actin stress fibre formation can be observed. These dynamic reorganizations of actin structure are probably mediated by regulatory proteins such as myosin [71], but the molecular mechanisms controlling F-actin dynamics during EMT remain to be elucidated. However, Gagat et al. proved that the stabilization of F-actin induced by overexpression of TPM1 (tropomyosin-1) led to a decrease in the percentage of the late apoptotic and necrotic cells in response to treatment epithelial cells line EA.hy926 with L-homocysteine thiolactone hydrochloride [72]. At the same time overexpression of TPM1 obtained by introducing an additional copy of the gene in the plasmid does not affect the cell migratory potential, although it has prevented cells from mobility reduction caused by L-homocysteine as in wound healing assay; after the 12 h culture the wound was almost completely repaired in case of cells with TPM1 overexpression, while for cells transfected with the empty plasmid the edges of the wound were still clearly visible. The study also outlined the conclusions regarding *α*-catenin contribution to the suppression of actin polymerization within intracellular junctions. This information is consistent with the data obtained by Yamada et al. which suggest that there is no possibility of simultaneous bounding of the E-cadherin-*β*-catenin complex and actin filaments by *α*-catenin [73, 74]. Furthermore, researchers on the base of *β*-catenin and the ZO-1 fluorescence signal reinforcement in cells with tropomyosin-1 upregulation suggested that F-actin increases the number of cell-cell junctions [72].

The process of actin microfilaments creation and decomposition is controlled by actin-binding proteins (ABP). They can influence the dynamics of the polymerization by releasing the monomers of actin from the protein complexes (actin/profilin), blocking the ends of the microfilaments (gelsolin, vilin, and fragmin), branching (Arp 2/3 complex), formation of new polymerization sites (cofilin), microfilaments bundles creation (fascin, fimbrin), and the stabilization of actin networks (formins) [75]. In turn, the binding of the monomeric or polymeric form by ABP is strictly controlled by intracellular protein signalling cascades. In response to internal (osmotic pressure, Ca^2+^ concentration) or external factors (growth factors) ABPs activate the GTPases of the Rho family. Rho GTPases are the main regulators of actin dynamics and controllers of actin rearrangement during EMT.

Many studies on the function of Rho proteins indicate their contribution to the creation of actomyosin-based structures and regulation of their contractility. In the wide range of mammalian cell cultures addition of activated Rho protein leads to enhanced accumulation of stress fibres [76]. This protein is also necessary to heal small incisions in the chicken embryo* in vivo*, which involves the contraction of an actin-based purse-string [77]. Rho action is additionally required for smooth muscle cells contractions [78]. These processes are mediated by ROCK (Rho-associated kinase), which phosphorylates light myosin chains and increases cell contractility [79]. In addition, Rho can increase the total amount of F-actin in the cell by promoting its polymerization, probably from new nucleic sites, rather than by their formation. However, this effect is varied for different cell cultures types [80]. As a result of Rho activation, ROCK interacts with the DIA1 (formin diaphanous 1) and promotes actin polymerization [81]. Rho-family GTPases control the actin cytoskeleton functioning in epithelial as well as in mesenchymal cells and seem to play an important role in both developmental and cancer-related EMT [44]. The activity of Rho GTPases is strictly controlled by GEFs (guanine nucleotide exchange factors), GAPs (GTPase-activating proteins), and GDIs (guanine nucleotide dissociation inhibitors) [82]. Due to the confirmed presence of both Rho GTPases and their activators, the contribution of these proteins to the polymerization of nuclear actin can also be deduced [83].

Both actin forms may play an important role in gene expression regulation. Monomeric form of actin was proved to bind with BRG1 (brahma-related gene 1), which is a part of BAF (BRG1-associated factor) chromatin remodelling complex and as its component can affect the availability of genes. In this case G-actin is involved in the proper interaction of the complex with chromatin [84]. On this basis, it can be concluded that the nuclear actin may regulate the access of transcription factors to particular DNA fragments, which results in controlling their expression [85]. Actin has also the ability to interact with all three RNA polymerases, and thus it is a part of the basal transcription machinery [86–88]. In this case, the form of the protein is yet to be elucidated; however, some evidence points to the involvement of the monomeric form [89]. Nevertheless, actin polymer may also take part in the transcription, as studies using actin polymerization inhibitors indicate that chemical inhibitors of polymerization process impair transcription [18, 90]. Furthermore, polymeric actin in association with NMI (nuclear myosin I) was proved to contribute to the polymerases I and II transcription [88, 90, 91] and intranuclear movement of interphase chromosomes [21] or their parts [22].

**Figure 4:**
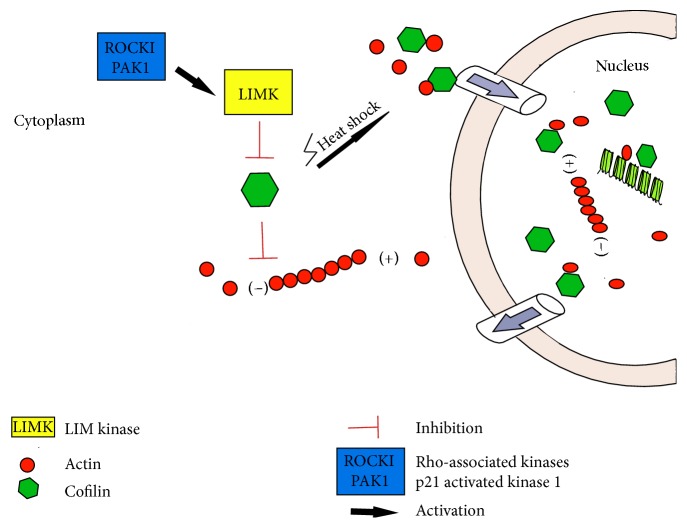
*Role of CFL1 in the response to stress conditions*. Under stress condition CFL1 translocate to the cell nucleus, where it can influence transcription. This takes place due to enhanced actin nuclear import and local depolymerization of F-actin.

Due to the involvement of both actin forms in processes occurring in the cell nucleus, their dynamics must be strictly controlled, and that may be the role of Rho proteins. Particularly, many of the ABPs present in the nucleus are downstream effectors for Rho GTPases and also take part in the regulation of gene expression. One such protein is cofilin, whose cytoplasmic activity is associated with the disassembly of actin filaments by promoting the F-actin depolymerization. Rho protein effectors like ROCKI and II or PAK1 (p21 activated kinase 1) are able to activate LIMK (LIM kinases) via phosphorylation. In turn, phosphorylation of the cofilin serine-3 rest by LIMK leads to the blockade of actin-binding ability of this protein, thus simultaneously stabilizing the actin cytoskeleton [92]. CFL-actin interactions probably play an important role in the cell nucleus, especially under stress conditions. As it was shown, the activity of factors such as heat shock or Latrunculin treatment leads to nuclear accumulation of actin, but also CFL [27, 93]. Furthermore, cofilin may influence gene expression, as the actin nuclear import enhances transcription and the whole process is regulated by CFL [29]. Moreover, cofilin may also contribute to transcription in direct way. Obrdlik and Percipalle showed that local depolymerization of F-actin caused by CFL1 is crucial for transcript elongation [94] (Figure 4).

Elevated levels of CFL1 expression were also associated with tumour progression and increased invasiveness in breast [95], urothelial [96], prostate [97], and gastric cancer [98]. which correlated with the presence of EMT markers. Haibo et al. proved that in the case of BGC-823 gastric cancer cells during TGF-*β*1-induced EMT the cofilin expression was elevated. Moreover, the silencing of CFL expression with siRNA inhibited the EMT process under the same inducing conditions. At the same time, with increased expression of cofilin, enhanced filopodia formation was observed. These results were consistent with changes observed in vivo. In nude mice xenograft model silencing of CFL1 expression again correlates with inhibited cancer progression and lack of EMT markers [98]. However, increased expression of CFL1 in tumours is not only related to the cytoplasmic region, but also to the cell nucleus. Results obtained by Hensley et al. point to a significant connection between EMT, CFL1 nuclear localization, and bladder cancer progression. Researchers have shown that progression of the disease correlates with the increased level of CFL in the nucleus. Furthermore, the samples from patients who died from bladder cancer have a statistically significant increase in expression of N-cadherin and nuclear cofilin while decreasing expression of E-cadherin compared to patients who survived [96]. These studies may suggest that CFL1 is involved in EMT not only by promoting the formation of invasive structures such as filopodia but also by controlling gene expression due to a regulation of actin organization in the nucleus.

In addition, other members of Ras superfamily are involved in the EMT process. For example, Rac protein was proved to stimulate lamellipodia formation, which was observed in the case of quiescent fibroblasts [99] or Nl E-115 neuroblastoma cells [100]. As the lamellipodia formation is driven by actin polymerization researchers also checked the impact of Rac on the F-actin formation. Machesky and Hall proved that Rac is able to stimulate the incorporation of fluorescently labelled actin monomers into filaments in lamellipodia [101]. Moreover, another Ras protein, Cdc42, is noted to be overexpressed in numerous cancer types [102–105]. That increase in the protein expression was associated with the higher migratory potential of cancer cells. These data are consistent with results obtained by Yamaguchi et al., who showed that activation of Cdc42 results in invadopodia formation and production or/and secretion of matrix metalloproteinases, the crucial elements for tumour cells invasion [106].

One of the most important ABPs with significant role in the EMT process is also SATB1 nuclear protein. Its main role is to bind DNA in sequences rich in AT pairs and therefore coordinate gene expression control through changes in chromatin-loop architecture. Data provided by Grzanka et al. showed that SATB1/F-actin complex may be detected at the border between condensed and decondensed chromatin compartments, which suggested its involvement in the transcription process [6]. Further studies conducted by these researchers indicated on SATB1/F-actin complex engagement in active cell death as the overexpression of SATB1 and CFL1 (enhanced actin transport to the nucleus) result in the increased percentage of apoptotic cells after geldanamycin treatment of MCF-7 breast cancer cell line [25]. It is also possible that SATB1 is involved in EMT process occurrence. As it was noted by Wan et al. SATB1 is overexpressed in BTCC (bladder transitional cell carcinoma) as well as in bladder cancer cell lines with high metastases creation potential. Moreover, increased SATB1 expression caused downregulation of E-cadherin through upregulation of its repressors (e.g., Slug, Snail). This indicates an important role of SATB1 in cancer progression through EMT promotion. Furthermore, the study on SATB1 expression in prostate cancer (PC) recently conducted by Honggang et al. confirms these assumptions. Also in this case increase in SATB1 expression was observed in PCs samples derived from patients and cell lines and the protein accumulated mainly in the nucleus [107]. In addition, overexpression of SATB1 leads to enhanced cell proliferation and migratory potential. SATB1 silencing was also connected with the increase in E-cadherin and decrease in vimentin expression (Figure 2).

It was also shown that the occurrence of EMT is associated with tumour cells resistance to chemo- or radiotherapy [108] and the survival rate of CSCs after conventional treatment is higher than for non-CSCs [109]. It may be even suggested that in the case of cells exhibiting EMT-associated features conventional therapies are connected more with selection rather than elimination of cells. This resistance has been linked to increased expression of antiapoptotic proteins, more effective drug efflux due to higher levels of ATP-binding cassette transporters and slower proliferation rate of stem cells. Also, some EMT-inducing transcription factors have been linked with tumour escape from immunological surveillance by preventing the abnormal cells from being detected [110].

## 5. Future Perspectives

Many genes identified as performing an important role in the course of EMT are down- or upregulated. Some of them may potentially serve as the process markers [111]. Due to the significance of this phenomenon in the tumour progression, EMT and its associated proteins have become highly interesting as a target for therapeutic agents. In particular, since 90% of cancer-associated mortality is caused by metastases, there is an intensive search for methods to target or prevent them [112]. Also, micrometastases may cause tumour recurrence, which is common in the case of surgical intervention, radiotherapy, and chemotherapy [113, 114]. One of the proposed solutions is the usage of antibodies against receptors involved in EMT initiation. This approach was applied to block TGF-*β*-induced EMT. It turns out that the specific inhibitor of TGF-*β* receptor SB-431542 restrains the EMT process in pancreatic cancer cells [115]. However, the efficiency is limited, due to a variety of factors able to initiate epithelial-to-mesenchymal transition as it may be impossible to block them all at once. Still, this problem can be overcome by targeting intracellular signal transduction pathways. Although transcriptional factors are not preferred therapeutic targets, it has been demonstrated that pharmacological inhibition of Snail expression blocks the EMT induction by TGF-*β* [116]. Another possibility may be the immunization of T lymphocytes against the transcription factors associated with EMT [117]. As a result, it would be possible to destroy tumour cells by the patient's own immune system which, in combination with conventional therapy, could lead to the complete cure. The disadvantage of both of these solutions is the need to initiate therapy in the early stages of tumour development before the metastasis process occurs. In advanced tumours in which the cell's phenotype has already changed to mesenchymal EMT inhibition may causes only minor effects [118]. Therefore, in patients with advanced cancers, it is possibly more reasonable to target cells exhibiting mesenchymal phenotype. One of the main mesenchymal markers is vimentin. This natural compound derived from a plant* Withania somnifera* in the case of human lung and breast cancer was responsible for vimentin filaments disassembling. That effect was associated with decrease in cell migratory and invasive potential but also increased apoptosis rate [119]. What is important, the compound is harmless to normal vimentin-expressing cells. It is probably because of higher soluble vimentin expression in healthy cells [120]. The second possibility may be targeting of another main mesenchymal marker, N-cadherin. Tanaka et al. showed that the use of the monoclonal antibody against N-cadherin was associated with inhibition of growth and metastasis of prostate cancer [121]. As the N-cadherin is also expressed in normal tissues like heart or liver tissue the researchers checked an impact of N-cadherin expression loss in mice. The model animals showed no evidence of sudden death, pathological changes in heart tissue, or abnormalities in cardiac enzymes levels in serum even at high doses (40 mg/km). A good option is also the inhibition of genes associated with the invasive capacity of mesenchymal cells with miRNA. As it was shown by Shi and colleagues the specific miR-21 inhibitor, AC1MMYR2, in the case of gastric cancer, breast cancer, and glioblastoma cell lines leads to EMT markers reduction and inhibition of invasion [122]. It was an effect of E-cadherin upregulation and reduction in expression of mesenchymal markers. The main disadvantage of this approach is the possibility of converting one migration type into another, for example, amoeboid [118].

Paradoxically even more favourable than EMT blocking may be inhibiting of MET process. It will not allow cancer cells circulating in the system to settle in secondary side and thus create metastases. However, the problem may be necessity of life-long therapy carrying [118]. Also, the occurrence of MET process is still poorly understood [35]. The best-studied example is MET associated with kidney formation, but there are still few studies on its course in the case of a cancer [123]. As in nephrogenic MET FGFs (fibroblast growth factor) and FGFRs (fibroblast growth factor receptor) are crucial [124, 125]; it is possible that one of the potential therapeutic targets of MET in cancer may be FGFR2IIIc, which has been also identified as a key element of epithelial phenotype in bladder carcinoma [58]. Study showed that reduced FGFR2 expression correlates with higher survival rate in mice inoculated with bladder carcinoma cells. The high expression of FGFR2 was also associated with the epithelial phenotype. These findings suggest that MET plays key role in metastases creation and can be blocked by inhibition of epithelialization.

Despite targeting EMT or MET processes, positive effects may also bring the inhibition of gene expression overexpressed in cells undergoing epithelial-to-mesenchymal transition. One of such proteins is SATB1. One of the agents able to inhibit SATB1 expression are HMG-CoA (3-hydroxy-3-methyl-glutaryl-coenzyme A) reductase inhibitors commonly known as statins. In addition to the cholesterol lowering capacity, those drugs may prevent cancer [126]. Research conducted by Lakshminarayana Reddy et al. showed that in COLO205 cells this effect is achieved by the downregulation of SATB1 by statins in the dose- and time-dependent manner. Inhibition of SATB1 expression has been noted after treatment with popular hydrophobic statins, simvastatin and fluvastatin, which has not been reported for hydrophilic statins, s.a. pravastatin [127]. Downregulation of STAB1 was proven to be an effective strategy for the TN (triple negative) breast cancer MDA-MB-231 cell line. TN breast cancer is one of the most aggressive forms of breast cancer with very poor prognosis. However, after treatment of MDA-MB-231 cells with SATB1-decoy DNA cell proliferation was inhibited and a significant reduction in invasiveness and migratory potential was observed [127]. On the other hand, the opposite effect was noted for Jurkat cells, where SATB1 knock-out was associated with increased invasiveness. This effect is most likely due to the nuclear *β*-catenin accumulation and the consequent activation of the Wnt/*β*-catenin signalling pathway [128]. This suggests the unsuitability of SATB1 inhibition-based therapy for such cancer types as adult T cell leukemia. Therefore, the effectiveness of this method in particular cancer types must still be carefully investigated. Similar attempts have been made to determine the effect of inhibition of the LIMK1-ADF (actin depolymerization factor)/cofilin activity on colon cancer cells. For this purpose, DADS (diallyl disulfide) was used. Studies show that DADS in colon cancer cells inhibits ADF/cofilin phosphorylation by downregulating the expression of LIMK1 and consequently suppresses cellular proliferation and migration in both in vitro and in vivo conditions [129]. In addition, this compound was also effective in the case of breast cancer [130] and myeloid leukemia cell lines [131]. However, there is no agreement as to the general mechanism of action of the potential drug in cancer and further research is necessary.

In addition to the search for effective antimetastatic drugs, the equally important factor which increases the chances of survival of oncological patients will be more accurate diagnostic methods that will help in the application of the solutions presented in the article. Detailed knowledge about the stage of cancer development will help in choosing the right type of therapy. It should also be remembered that there is evidence of the possibility of metastases in certain cancer types without the EMT or MET processes occurring [132]. Thus, we strongly believe that the future of cancer therapy is a higher personalization of treatment based on detailed diagnostics.

## 6. Conclusions

Epithelial-to-mesenchymal transition and its reverse process MET were proven to be an inseparable element of the cancer progression. Generally, overexpression of EMT markers is connected with poor prognosis and high probability of metastasis [35]. The change in the phenotype of a cell to mesenchymal involves the acquisition of features such as invasiveness and migration potential. In turn, the movement of cells is possible due to changes in actin cytoskeleton reorganizations consisting of polymerization and disintegration of actin filaments. The dynamics of this process can be regulated by actin-binding proteins such as CFL1 or SATB1 in several ways [75]. Although actin does not contain a nuclear localization sequence, many ABPs comprise it. As it was proven both actin forms also occur in the cell nucleus, and the presence of actin in this cell compartment may influence the regulation of gene expression [6]. The EMT process is not only related to actin but also related to its associated proteins such as SATB1, CFL1, or Rho kinases (ROCKI and ROCKII), whose overexpression was noted in many cancer types. Due to the fact that 90% of cancer deaths are directly caused by metastases [112] many studies focus on the suppression of EMT or MET. Although in many cases satisfactory results have been already obtained both in vitro and in vivo, there is still no commercially available drug targeting one of these processes or its associated proteins. Also, we still do not have a 100% reliable method as all of the described approaches have some disadvantages. However, due to the possibility of significant reduction of cancer-related mortality, further studies are needed. However, it should also be remembered that EMT-targeting therapy must be coadministered with conventional therapy to eliminate the primary tumour site.
